# Complete Genome Sequence of Shewanella chilikensis Strain DC57, Isolated from Corroded Seal Rings at a Floating Oil Production System in Australia

**DOI:** 10.1128/MRA.00584-20

**Published:** 2020-09-17

**Authors:** Silvia J. Salgar-Chaparro, Genis Castillo-Villamizar, Anja Poehlein, Rolf Daniel, Laura L. Machuca

**Affiliations:** a Curtin Corrosion Centre, WA School of Mines: Minerals, Energy, and Chemical Engineering, Curtin University, Bentley, Western Australia, Australia; b Genomic and Applied Microbiology and Göttingen Genomics Laboratory, Institute of Microbiology and Genetics, Georg August University of Göttingen, Göttingen, Germany; c Línea Tecnológica Biocorrosión, Corporación para la Investigación de la Corrosión, Piedecuesta, Santander, Colombia; University of Maryland School of Medicine

## Abstract

Here, we describe the genome of Shewanella chilikensis strain DC57, a facultatively anaerobic bacterium isolated from corroded seal rings at a floating oil production system in Australia. The genome of strain DC57 has a size of 4.91 Mbp and harbors 4,178 predicted protein-encoding genes.

## ANNOUNCEMENT

Shewanella chilikensis is a facultatively anaerobic, Gram-negative, and rod-shaped bacterium (1). Members of the genus *Shewanella* have been reported to be associated with microbiologically influenced corrosion (2–6). *Shewanella* spp. have the ability to use a variety of electron acceptors, including nitrate, thiosulphate, and iron oxides (7), which indicates that these microorganisms can cause corrosion by different mechanisms.

*S. chilikensis* strain DC57 was isolated from corroded seal rings at a floating oil production system located in waters on the North West Shelf of Western Australia. Corrosion products were collected and inoculated in anaerobic phenol red broth medium (8). After positive growth in tubes incubated at 40°C, the culture was plated onto phenol red agar and incubated in anaerobic jars with AnaeroGen sachets (Oxoid). DC57 was purified using the streaking method until an axenic culture was obtained, as determined by microscopy. Single colonies were transferred to phenol red broth medium, and DNA was extracted using the DNeasy PowerSoil kit (Qiagen). Extracted DNA was sequenced with a combination of sequencing platforms. For Illumina sequencing, the library was prepared with the Nextera XT DNA sample preparation kit, and paired-end reads were generated on the MiSeq platform using the MiSeq reagent kit v3-600, as recommended by the manufacturer (Illumina, San Diego, CA, USA). For Nanopore sequencing, genomic DNA was prepared using the ligation sequencing kit 1D (SQK-LSK109) without any size selection. Sequencing was performed with the MinION Mk1B device and a SpotON flow cell R9.4, as recommended by the manufacturer (Oxford Nanopore Technologies, Oxford, UK). Base calling was performed using Albacore v2.3.1. Quality filtering of the reads was performed with fastp v0.19.4 (9), which resulted in 3,370,098 short reads (Illumina) with an average length of 245 bp, and 654,567 long reads (Nanopore) with an average length of 1,813 bp. A hybrid assembly strategy using Unicycler v0.4.7 (10) was applied to perform a *de novo* genome reconstruction, with overlap removal, circularization, and rotation. The assembly was validated with Bandage v0.8.1 (11). Default parameters were used for all software unless otherwise specified.

The complete genome of DC57 comprises a single circular chromosome (4,910,425 bp) with an overall GC content of 52.35% and 162-fold coverage. Annotation was performed with the NCBI Prokaryotic Genome Annotation Pipeline (PGAP) v4.10 (12), which predicted 4,434 genes, including 104 tRNA genes, 25 rRNA genes, 4 noncoding RNA genes, 4,178 genes encoding proteins with predicted functions, and 123 genes encoding hypothetical proteins. Classification was performed by calculating the average nucleotide identity (ANI) with the Python module for ANI analyses (pyANI) v0.2.7 (13). This analysis revealed that DC57 is closely related to *S. chilikensis* strain JC5 (GenBank accession number NZ_NIJM00000000.1) with an ANI value of 98.86%.

The genome analysis revealed the presence of the metal reduction pathway (MTR), two pathways for nitrate reduction (NAP and NAR), and genes for thiosulfate reduction (*phsA* and *glpE*), which could be related to the corrosive potential of the strain.

### Data availability.

The genome sequence of Shewanella chilikensis strain DC57 was submitted to GenBank under accession number CP045857. The raw reads were deposited in the NCBI SRA database under accession numbers SRR11492373 and SRR11492374.
